# Prevalence and Risk Factors of Occupational Health Hazards among Health Care Workers of Northern Saudi Arabia: A Multicenter Study

**DOI:** 10.3390/ijerph182111489

**Published:** 2021-10-31

**Authors:** Ashokkumar Thirunavukkarasu, Khaloud Amash Hossin Alrawaili, Ahmad Homoud Al-Hazmi, Umar Farooq Dar, Bashayer ALruwaili, Ayesha Mallick, Farooq Ahmed Wani, Amnah Ibrahim E Alsirhani

**Affiliations:** 1 Department of Community and Family Medicine, College of Medicine, Jouf University, Sakaka 72388, Saudi Arabia; ahhazmi@hotmail.com (A.H.A.-H.); ufdar@ju.edu.sa (U.F.D.); bfalrwili@ju.edu.sa (B.A.); amhaseeb@ju.edu.sa (A.M.); 2 Department of Public Health, Tarif Al-Salhiya Health Center, Ministry of Health, Turaif 3753, Saudi Arabia; khulud-rwaili@hotmail.com; 3 Department of Pathology, College of Medicine, Jouf University, Sakaka 72388, Saudi Arabia; fawani@ju.edu.sa; 4 Department of Family medicine, Ministry of Health, Tabuk Region, Tabuk City 47512, Saudi Arabia; amnahalsirhani@gmail.com

**Keywords:** occupational health hazards, risk factors, health care workers, work-related stress

## Abstract

Health care workers (HCWs) working in different health care facilities are exposed to many hazards, especially during the COVID-19 pandemic. This questionnaire-based cross-sectional study aimed to assess the prevalence, pattern, and risk factors of occupational health hazards faced by 438 randomly selected HCWs from northern Saudi Arabia. The HCWs are commonly exposed to needle stick injuries (34.5%) under the biological hazards category; and work-related stress (69.6%) under the non-biological hazards categories. The significant associated factors were work setting (ref: Primary Health Center: Adjusted OR (AOR) = 2.81, 95%CI = 1.21–4.59, *p* = 0.017), smoking status (ref.: non-smoker: AOR = 1.73, 95%CI = 1.03–2.91, *p* = 0.039), and mean sleeping duration per day (AOR = 1.22, 95%CI = 1.04–1.43, *p* = 0.014) for biological, and smoking status (ref: non-smoker: AOR = 2.16, 95%CI = 1.09–3.29, *p* = 0.028), and mean sleeping duration per day (AOR = 1.35, 95%CI = 1.07–1.70, *p* = 0.013) for non-biological categories. This study revealed several risk factors and occupational health hazards that HCWs are exposed to during their work time. Periodic training and follow-up assessments regarding bio-safety measures for the HCWs should be implemented. Finally, future explorative studies are warranted on the feasibility of implementing rotation-based postings for the HCWs in different health care settings.

## 1. Introduction

A health care facility is a workplace that aims to give care to the patients in terms of diagnosis, treatment, and prevention activities [1]. Personnel working in any health care facility are considered as health care workers (HCWs) [2]. Health care facilities worldwide employ over 59 million workers, as stated by the World Health Organization (WHO). In the Kingdom of Saudi Arabia (KSA), approximately 424,000 workers are employed in health care settings, including Saudis and other nationals [3]. The WHO classified the HCWs into a wide range of categories, primarily as medical practitioners (general and specialty), dentists, nursing and midwifery professionals, pharmacists, and other allied health professionals [4]. Health care personnel working in different health care facilities are exposed to the risk of a wide array of hazards at work, including infections due to sharps injuries, harmful exposures to radiation and dangerous drugs, injuries, physical violence, and mental stress [5,6,7,8]. Though biological hazards such as infections are commonly recognized worldwide, the non-biological hazards and their importance are generally ignored [9,10]. The health hazards faced by the HCWs due to bacterial and viral respiratory infections (biological hazard), including COVID-19, are higher than the general population [11,12]. Amnesty International reported that globally more than 7000 HCWs have lost their life due to COVID-19 [13]. Besides the COVID-19 infections, the HCWs are more likely to develop other serious respiratory tract infections such as latent tuberculosis infections and symptomatic tuberculosis [14,15].

The working conditions of HCWs are essentially linked to the quality of care given to the patients. The exact working conditions, including shift duties, psycho-social factors, team management, working hours, and culture are associated with their health and safety outcomes [16,17,18]. In addition, HCWs are considered immune to becoming sick by the general population. However, in reality, the scenario is entirely different as reported by previous studies [2,19,20,21,22,23]. The protection of the health of the HCWs has the benefits of improving public health as they contribute to 10 to 18% of the workforce of any country [3,24]. Even though it is achievable to decrease occupational hazards among HCWs, they continue to face biological and non-biological health hazards at the workplace worldwide [25,26].

In the KSA, the ministry of health (MOH) implemented a uniform policy to follow in all public and private health care facilities to provide a safe and healthy environment for the HCWs, patients, and visitors. Each type of health care facility enacted its vision, mission, and policies in alignment with the policy of the MOH [27]. The continuous assessment of occupational health hazards faced by the HCWs is essential for strengthening the public health care system in a country [17,24]. This can be achieved by identifying the current prevalence and risk factors of health hazards acquired by the HCWs due to their workplace [24,26]. A study conducted by Abdulmageed et al. in 2018 assessing the risk factors for biological hazards in tertiary care hospitals found that significant risk factors for needle stick injuries were extended workload and shift duties [20]. Another study performed in Uganda by Ndejjo et al. mentioned that work-related stress, type of health care facilities, and improper personal protective measures were significantly associated with a higher risk of developing biological and non-biological health hazards [21]. A study by Alenzi et al. in the KSA during the COVID-19 pandemic stated that stress and anxiety were significantly higher among the unmarried, elderly, nurses, and workers in radiology [28]. The literature review of the present study found only a limited number of studies from KSA on the occupational hazards of HCWs that covered biological and non-biological hazards and their prevalence and risk factors. Those published studies are focused on needle injuries, awareness of occupational infections hazards, and/or are conducted in single health care settings. Hence, this study was planned to cover all the WHO categories of HCWs from all health care settings [4]. The present study aimed to assess the prevalence, pattern, and risk factors of occupational health hazards faced by the HCWs working in different health care settings in northern Saudi Arabia.

## 2. Materials and Methods

### 2.1. Study Design and Setting

This analytical cross-sectional study was executed from April 2021 to September 2021 among the HCWs working in different health care facilities in the northern border province of Saudi Arabia. The northern border province is situated in northern Saudi Arabia with an area of 127,000 km^2^ and a population estimated to be around 300,000. This province is further subdivided into four regions: Ar’ar, Turaif, Rafha, and Aluwaikilah. In the KSA, health care to the public is delivered through four levels: primary health centers (PHC), general hospitals, specialty hospitals, and medical cities (apex hospitals). Currently, there is no medical city in the northern border region. The PHC provides the services related to essential curative, prevention, and referral to a higher health center (general or specialty). In addition to the above-mentioned services, the general hospital also provides services related to outpatient clinics, laboratory, radiological investigations, and obstetrics and gynecology (delivery unit), while the specialty hospitals offer the most sophisticated health care services involving all surgical and medical specialties departments.

### 2.2. Sample Size Estimation

The sample size was calculated based on the formula z^2^ pq/e^2^. In this formula, z is 1.96 at a 95% confidence interval, p is expected proportion, q is 1–p, e alpha level at 0.05 (margin of error at 5%). The following factors considered while calculating the sample size were expected proportion (p) as 50%, power of the study as 80%, and confidence interval as 95%. There was no published study identified from the extensive study of the literature in the KSA on this subject covering entire health care settings. Hence, the research team took the expected proportion (p) as 50% to get the maximum sample size. Considering the above factors with the anticipated 20% non-response rate, the total sample size was calculated as 480 (384 × 100/20).

### 2.3. Sampling Method

A multi-stage probability proportional to size (PPS) sampling method was used to select the study population. In the first stage, one specialty hospital, one general hospital (was selected randomly by lot method from all the list of hospitals), and all the PHCs were selected. The list of health care facilities and the number of HCWs working in each selected health care facility were obtained from concerned authorities. The required number of study participants from each health care facility and each subgroup of HCWs were selected based on PPS in the following stages. Finally, the systematic random sampling method was used to select the required number of study participants from health care facilities based on their employment number.

### 2.4. Ethical Consideration

The Local Committee for Bioethics (LCBE) of Jouf University has issued the ethical clearance to conduct this study (LCBE No: 10-08-41).

### 2.5. Inclusion and Exclusion Criteria

The study included all the HCWs classified by the WHO, such as medical and dental practitioners, nursing and midwifery, pharmacists, laboratory technicians, and other allied health feld professionals. The study participants included were only the HCWs working in the government sectors. The HCWs on vacation and the HCWs working in private health care settings, such as those working in private clinics, hospitals, medical laboratories, and pharmacies, were excluded from the sampling frame.

### 2.6. Data Collection

#### 2.6.1. Data Collection Procedure

After the necessary approval, the data collection process was initiated. The principal and coinvestigators communicated with the selected study participants to obtain their availability at their workplace. The measures were taken not to communicate with the participants for data collection during their night duty and post duty off days. The selected participants were briefed about the study and their willingness to participate was requested through informed consent. The research team tried to communicate with the selected participants three times a month. The participants who were not willing to participate and/or those whom the research team could not contact were considered as non-respondent. The research team followed strict COVID-19 prevention protocols of the Ministry of Health, KSA, during the data collection process.

#### 2.6.2. Tool for Data Collection

A standardized, validated, and self-administered questionnaire was given to the selected participants. This questionnaire was prepared by the research team with experts’ opinions based on the available open-source pieces of literature [5,21,25,29]. The structured questionnaire was tested for validity (content and face) as well as reliability. The content validity of the questionnaire was assessed by two independent experts from the public health and occupational medicine specialties. Then, the pilot study was performed among 30 different categories of the HCWs to test face validity. There were no missing data found, and all the participants agreed that the questionnaire was simple and easy to understand. The reliability test was performed by using Cronbach’s alpha analysis, and the alpha value of the reliability test was found to be 0.83. Hence, the research team proceeded to collect data for the main study with this structured, self-reported questionnaire. It consisted of two parts.

Part 1: This inquired about the study participants’ socio-demographic and health-related activities such as age, gender, marital status, sleep duration, smoking habit, work experience, health care setting type, and HCW category. The participants were asked to mention their age in years. In addition, the participants responded about their sleep duration for the question, “What was your average sleep duration during the past one month”?. All other variables were categorized as per the coding sheet. These factors are independent variables and were used for the risk factors identification.

Part 2: This section collected the details related to occupational health hazards faced by the HCWs. Participants were asked to respond to ten types of occupational hazards, e.g., “Have you ever experienced and/or exposed any of the following hazards listed below during your working time in the health care facility in the last twelve months?” Of the ten types of health care hazards, the first five items were related to biological, and the remaining five were related to non-biological health hazards. Part 2 responses were considered for the outcome variable analysis. This survey assessed the work-related stress in the past 12 months under the non-biological health hazards category through two questions. The first question was “How frequently have you perceived that you were unable to cope with the essential things?”; and the second was “How frequently have you felt that you could not do all the duties that you had to do?”. The participants responded on a 5-point Likert scale ranging from never to very often and scored from 1 to 5 based on the response. A participant with a total score of 5 and above from the above two questions was perceived to have work-related stress.

### 2.7. Statistical Analysis

Descriptive statistics of the present study are presented as frequency (numbers; *n*), the percentage for qualitative variables, and mean ± standard deviation (SD) for quantitative variables. The logistic regression analysis was performed to identify the association between occupational health hazards and socio-demographic characteristics. The significant associated factors were considered as risk factors. The outcome variable of the present study was measured by the exposed occupational health hazard (yes/no). Initially, the univariate analysis was executed to compare each independent variable with the outcome variable, followed by binomial logistic regression through the “enter” method. In this regression analysis, adjusted independent variables were age, gender, marital status, nationality, work setting, HCW category, work experience, smoking status, and sleep hours per day. In the logistic regression analysis, odds ratios (ORs) with 95% confidence intervals (CIs) were calculated. A confidence interval that did not include a null value of one and a *p*-value of less than 0.05 was considered statistically significant. The Statistical Package for Social Sciences (SPSS) software version 20 was used for data entry and analysis.

## 3. Results

This research was carried out among different HCWs working in different health care facilities in the northern border province of Saudi Arabia. Out of 480 selected participants, the number of respondents was 438, with a response rate of 91.3%.

Table 1 presents the socio-demographic and lifestyle-related characteristics of the sample population. Of the 438 study participants, 60% were males, and 40% were females with the mean age of 38.2 ± 8.8. The majority of the sample studied were married (63.7%), Saudi nationals (63%), and had never been a smoker (55%). Of the sample studied, 41.6% of participants were working at different PHCs, and the mean duration of sleeping of the participants was 6.9 ± 1.4 h per day.

The pattern of occupational health hazards (biological and non-biological) faced by the HCWs in this study is presented in Table 2. The common biological hazards faced by the study population were needle stick injuries (34.5%) and infections due to airborne diseases (31.1%). The common non-biological hazards faced by the study population were work-related stress (69.6%), followed by physical, psychological, sexual, and/or verbal abuse (52.7%), and musculoskeletal problems such as muscle aches/strains/sprains (39.7%).

Of the sample studied, 300 (68.5%) participants were exposed to one or more biological hazards, while 384 (87.7%) participants faced one or more non-biological hazards. Table 3 presents biological health hazards and their association with socio-demographic and lifestyle-related characteristics. In the univariate analysis, the characteristics that were significantly associated with biological hazards were age (OR = 1.06, 95% CI = 1.03–1.08, *p* < 0.01), gender (ref: male: OR = 0.62, 95%CI = 0.44–0.94, *p* = 0.023), work setting (ref: PHC: OR = 3.45, 95%CI = 2.04–4.82, *p* < 0.01), smoking status (ref: non-smoker: OR = 1.69, 95%CI = 1.12–2.56, *p* = 0.013), and mean sleeping duration per day (OR = 1.20, 95%CI = 1.04–1.40, *p* = 0.014). The multivariate analysis revealed only the following three characteristics were significantly associated after being adjusted with other independent variables: work setting (ref: PHC: Adjusted OR (AOR) = 2.81, 95%CI = 1.21–4.59, *p* = 0.017), smoking status (ref: non-smoker: AOR = 1.73, 95%CI = 1.03–2.91, *p* = 0.039), and mean sleeping duration per day (AOR = 1.22, 95%CI = 1.04–1.43, *p* = 0.014).

Non-biological health hazards and their association with socio-demographic and lifestyle-related characteristics are presented in Table 4. The univariate analysis found that non-biological hazards were significantly associated only with the mean sleeping duration per day (OR = 1.36, 95% CI = 1.08–1.70, *p* = 0.008). The multivariate analysis revealed the following two characteristics were significantly associated after being adjusted with other independent variables: smoking status (ref: non-smoker: AOR = 2.16, 95%CI = 1.09–3.29, *p* = 0.028), and mean sleeping duration per day (AOR = 1.35, 95%CI = 1.07–1.70, *p* = 0.013).

## 4. Discussion

“World patient safety day” is marked every year on 17 September by the WHO to promote patients’ safety through different programs collaborating with its all-key stakeholders. Patients’ safety is considered one of the global public health priorities. The WHO observed the World Patient Safety Day, 2020, with the theme of “Health worker safety: a priority for patient safety.” This reiterates the need for a safe and healthy working condition for HCWs as an essential component for protecting patient safety, especially during the COVID-19 pandemic [30]. This can be achieved by assessing the hazards faced by the HCWs. Hence, this study was planned to assess the prevalence, pattern, and risk factors of occupational health hazards faced by HCWs.

The present study found that nearly one-third (34.5%) of participants experienced a needle stick injury during their work time. A study completed by Samia G et al. among HCWs in a University hospital, Jeddah in 2018 [20] found results similar to the present study. In contrast, some other studies performed in KSA by Omar et al. in Al-Medina City and Jahan S in Buraidah City have found a lower prevalence of needle stick injuries [23,31]. In their studies, the prevalence of needle stick injury was 24% and 22.5%, respectively. Interestingly, a study conducted in Alexandria, Egypt, found a remarkably high prevalence (67.9%) of needle stick injuries among the HCWs [32]. These differences in prevalence could be explained due to study settings such as types of inclusion of health care facilities and the types of HCWs. This study sampled all the HCWs of different types of health care facilities.

The HCWs are exposed to several bacterial and viral respiratory infections, including serious illnesses like tuberculosis, and this scenario has worsened due to the COVID-19 pandemic [14,33]. In the COVID-19 pandemic context, the HCWs are considered as high risk and vulnerable population to get active infections and act as a carrier of these infections to other patients, locations, and families. Hence, it is essential to protect the HCWs from getting COVID-19 and other serious airborne infections [33,34]. The current study results revealed that 31.1% of participants were exposed to one or more airborne-related infections. A systematic review conducted by Kent A et al. also found that the prevalence of airborne infections among health care workers ranges from 15 to 41% [35]. A study conducted in Ethiopia has shown that more than one-third of the HCWs were positive for latent tuberculosis [14]. Another study performed in the KSA revealed that COVID-19 infections among the HCWs were higher than in the general population [36].

The present study revealed that nearly two-thirds (68.5%) of the study population were exposed to one or more biological health hazards such as needle stick injuries and so on. These biological health hazards were significantly higher among the HCWs working in higher centers (tertiary/referral/speciality hospitals) than the HCWs of PHCs (AOR = 2.81, 95%CI = 1.21–4.59, *p* = 0.017). A study conducted by Ndejjo et al. in 2015 also found that biological health hazards are significantly associated with the type of health care facilities (AOR = 2.21, 95% CI = 1.02–4.78, *p* = 0.043) [21]. It is noteworthy to mention again that in the KSA, high-risk procedures, critical care, and care related to COVID-19 (pulmonology, infectious diseases, etc.) are generally performed at the specialty hospitals, and PCHs are equipped to provide essential health services only. This could explain why HCWs working at higher centers reported a significantly higher risk of developing biological health hazards in the present study. Similar to the present study, Macintyre et al. also stated that the HCWs performing high-risk procedures had a significantly higher rate of developing respiratory infections and other laboratory-associated bacterial and viral infections [37].

In a health care setting, the work-related stress of the HCWs may have a significant negative impact on the effective health care delivery to the patients [38]. In the present study, 69.6% of participants reported work-related stress. A study conducted by Alenzi H et al. in 2020 also found similar findings to the current study. In their study, self-reported stress and anxiety were present among 68.5% of HCWs [28]. In contrast, a study by Makhaita H et al. in Dammam, KSA, reported a relatively lower level (45.5%) of work-related stress [39]. The higher prevalence of stress in the present study and the study done by Alenzy H is mainly due to the COVID-19 pandemic, as both the studies were conducted during the COVID-19 pandemic. Globally, the increase in stress, burnout, and anxiety among the HCWs during the COVID-19 pandemic is a noted fact [40,41,42].

The current study depicted that nearly half (52.7%) of the participants faced some form of abuse/violence such as physical, psychological, sexual, and/or verbal abuse. Similar to the present study, some studies conducted in KSA by Harthi MM et al. in 2019 [43], and Alsaleem AS et al. in 2018 [44] found some form of abuse/violence prevalence as 47.8% and 57.5%, respectively.

The present study revealed a remarkably high proportion (87.7%) of participants who faced one or more non-biological hazards. These hazards were significantly associated with smoking status (AOR = 2.16, 95%CI = 1.09–3.29, *p* = 0.028), and mean sleeping duration per day (AOR = 1.35, 95%CI = 1.07–1.70, *p* = 0.013). Similarly, an association between smoking and non-biological hazards in the workplace was found by some other authors [45,46]. Interestingly, the present study findings revealed that smokers have a higher risk of developing biological health hazards, in addition to non-biological health hazards. Similar to the present study results, a survey conducted by Weldesamuel et al. in 2019 among the Ethiopian HCWs revealed that cigarette smokers had a higher prevalence of biological health hazards like needle stick injuries and sharp injuries [47]. These interesting associations between smoking and both types of health hazards could be explained by the different theories and models explored in the past. Firstly, cigarette smoking addiction leads to the alteration of neuroendocrine mechanisms (hypothalamic-pituitary-adrenal axis) and activation of the brain stress system [48,49]. This theory is supported by a study by Stubbs B et al. in 41 countries. In their study, the perceived stress was significantly higher among smokers in most of the countries [50]. The significant association between smoking and biological health hazards could be explained due to the higher rate of tremors and coughs among smokers [51]. Hence, the possibility of needle stick injuries and exposure to airborne infections are higher among smokers.

Similar to the present study findings, a systematic review and meta-analysis performed by Uehli et al. found that workers with sleep problems had a significantly higher risk of developing non-biological hazards such as injuries than workers without sleep problems [52].

The present study did not find an association between different HCW categories and both biological and non-biological health hazards. Similarly, a survey by Ndejjo et al. also revealed no significant association between the type of HCW category and the hazards faced by them [21]. In contrast, some authors found that HCWs, especially nurses, were significantly associated with needle stick injuries and exposure to respiratory infections, including COVID-19 [28,36]. This striking difference could be explained due to the difference in the inclusion of health care facilities. The present study included all three levels of health care facilities, while latter studies were done at tertiary care centers. Additionally, by using the PPS method, this study recruited 41.6% of participants from the PHCs, and unlike tertiary care hospitals, only essential health care services are provided at the PHCs.

Even though the research team made the best effort to find the prevalence of occupational hazards faced by HCWs with the standard methodology, certain limitations need to be noted while interpreting the results of this study. Firstly, this study is cross-sectional and does not find the temporal association between the risk factors and outcome variables. Secondly, the study’s data were self-reported, and it has limitations related to self-reported studies such as being subjective based, exaggerated reports, and recall bias. Furthermore, the present study covered the self-reported hazards only in broader aspects. Hence, it is recommended to do more multi-center explorative and prospective studies involving each sub-category of health hazards faced by the HCWs.

## 5. Conclusions

The present study revealed that HCWs faced several occupational health hazards during their work time. This study also found several risk factors for biological and non-biological health hazards such as working in higher-level centers, smoking, and lack of sleep. To mitigate the sleep problems of the HCWs, the health care facilities and policymakers must make a judicious work schedule of night-shift duties. Measures should be established at the health care facilities to increase compliance with the regulations instituted by the concerned authorities, such as infection control, bio-safety rules, and regulations that protect the HCWs from acquiring airborne infections. The current study results suggest the implementation of smoking cessation programs along with necessary legal measures at all health care facilities. Periodic training for bio-safety measures, well-defined abuse reporting procedures, and regular follow-up assessments must be instituted. Finally, future explorative studies are warranted on the feasibility of implementing rotation-based postings for the HCWs in different health care settings.

## Figures and Tables

**Table 1:** Socio-demographic and lifestyle characteristics of study population (*n* = 438).

Characteristics	Frequency (*n*)	%
Age (mean ± SD)	38.2 ± 8.8	
Gender		
Male	263	60
Female	175	40
Marital status		
Single	117	26.7
Married	279	63.7
Widowed/Divorced	42	9.6
Nationality		
Saudi	276	63
Non-Saudi	162	37
Work setting		
Primary health center	182	41.6
General hospital	161	36.8
Tertiary care/Specialty hospital	95	21.7
HCWs category		
Practitioner (Medical and Dental)	176	40.2
Nursing and Midwifery	127	29
Lab technicians	68	15.5
Others (all remaining WHO categories)	67	15.3
Work experience		
Less than 5 years	131	29.9
5 to 10 years	157	35.8
>10 years	150	34.2

**Table 2:** Pattern of occupational health hazards faced by the study participants (*n* = 438).

Variable	Number	%
**Biological**		
Needlestick injury	151	34.5
Infections due to airborne such as influenza, COVID-19, tuberculosis, etc.	136	31.1
Direct contact with contaminated specimens/biohazardous materials	127	29.0
Cuts/lacerations	85	19.4
Cross-contamination from soiled materials	52	11.8
**Non-Biological**		
Work-related stress	305	69.6
Physical, psychological, sexual, and/or verbal abuse	231	52.7
Musculoskeletal injuries such as muscle aches/strains/sprains	174	39.7
Slips, trips, and falls	116	26.5
Others (noise, burns, and chemical spills)	97	22.1

**Table 3:** Biological health hazards and their association with socio-demographic and lifestyle-related factors.

Characteristics	Total Sample (*n* = 438)	Health Hazard Status		Univariate Analysis of Health Hazards No vs. Yes		Multivariate Analysis of Health Hazards * No vs. Yes	
		No (*n* = 138) *n* (%)	Yes (*n* = 300) *n* (%)	OR (95% CI)	*p*-Value	Adjusted OR (95% CI)	*p*-Value ^**^
Age (mean ± SD)	38.2 ± 8.8			1.06 (1.03–1.08)	<0.01 ^**^	1.02 (0.98–1.07)	0.3
Gender							
Male	263	72 (27.4)	191 (72.6)	Ref	0.023 ^**^	Ref	0.73
Female	175	66 (37.7)	109 (62.3)	0.62 (0.44–0.94)		1.10 (0.63–1.91)	
Marital status							
Single	117	32 (27.4)	85 (72.6)	Ref		Ref	
Married	279	96 (34.4)	183 (65.6)	0.72 (0.45–1.12)	0.17	0.57 (0.34–1.24)	0.39
Widowed/Divorced	42	10 (23.8)	32 (76.2)	1.21 (0.53–2.73)	0.56	0.76 (0.61–1.15)	0.55
Nationality							
Saudi	276	95 (34.4)	181 (65.6)	Ref	0.09	Ref	0.29
Non-Saudi	162	43 (26.5)	119 (73.5)	1.45 (0.95–2.23)		1.36 (0.82–2.26)	
Work setting							
Primary health center	182	61 (33.5)	121 (66.5)	Ref		Ref	
General hospital	161	51 (31.7)	110 (68.3)	2.24 (1.37–3.64)	0.01 ^**^	2.00 (1.10–3.65)	0.023 ^**^
Tertiary care/Specialty hospital	95	26 (27.4)	69 (72.6)	3.45 (2.04–4.82)	<0.01 ^**^	2.81 (1.21–4.59)	0.017 ^**^
HCWs category							
Practitioner (Medical and Dental)	176	58 (33.0)	118 (67)	Ref		Ref	
Nursing and Midwifery	127	41 (32.3)	86 (67.7)	1.03 (0.63–1.68)	0.9	1.19 (0.67–2.13)	0.55
Lab technicians	68	19 (27.9)	49 (52.7)	1.12 (0.63–2.13)	0.64	1.28 (0.66–2.50)	0.47
Others (all remaining WHO categories)	67	20 (29.9)	47 (70.1)	1.27 (0.69–2.35)	0.45	1.13 (0.58–2.23)	0.73
Work experience							
Less the 5 years	131	62 (47.3)	69 (52.7)	Ref		Ref	
5 to 10 years	157	45 (28.7)	112 (71.3)	1.09 (0.69–1.71)	0.72	0.97 (0.59–1.58)	0.89
>10 years	150	31 (20.7)	119 (79.3)	1.34 (0.78–2.3)	0.3	1.55 (0.86–2.80)	0.19
Smoking status							
Non-smoker	241	88 (36.5)	153 (63.5)	Ref	0.013 ^**^	Ref	0.039 ^**^
Daily/Rarely	297	150 (50.5)	147 (49.5)	1.69 (1.12–2.56)		1.73 (1.03–2.91)	
Sleeping hours per day (mean ± SD)	6.9 ± 1.4			1.20 (1.04–1.40)	0.014 ^**^	1.22 (1.04–1.43)	0.014 ^**^

^*^ Variable(s) entered for logistic regression analysis: age, gender, marital status, nationality, work setting, HCWs category, work experience, smoking status, sleep hours per day, ^**^ *p* value less than 0.05 was considered as statistically significant.

**Table 4:** Non-biological health hazards and their association with socio-demographic and lifestyle-related factors.

Characteristics	Total Sample (*n* = 438)	Health Hazard Status		Univariate Analysis of Health Hazards No vs. Yes		Multivariate Analysis of Health Hazards No vs. Yes	
		No (*n* = 54) *n* (%)	Yes (*n* = 384) *n* (%)	OR (95% CI)	*p* Value	Adjusted OR (95% CI)	*p* Value ^**^
Age (mean ± SD)	38.2 ± 8.8			0.98 (0.95–1.01)	0.18	0.96 (0.90–1.02)	0.15
Gender							
Male	263	37 (14.1)	226 (85.9)	Ref	0.18	Ref	0.09
Female	175	17 (9.7)	158 (90.3)	1.52 (0.83–2.20)		1.93 (0.90–3.14)	
Marital status							
Single	117	12 (10.3)	105 (89.7)	Ref		Ref	
Married	279	38 (13.6)	241 (86.4)	0.73 (0.56–1.23)	0.36	0.73 (0.35–1.54)	0.41
Widowed/Divorced	42	4 (9.5)	38 (90.5)	1.09 (0.83–1.57)	0.89	0.88 (0.68–1.30)	0.84
Nationality							
Saudi	276	30 (10.9)	246 (89.1)	Ref	0.23	Ref	0.64
Non-Saudi	162	24 (14.6)	138 (85.2)	0.70 (0.59–1.25)		0. 85 (0.44–1.66)	
Work setting							
Primary health center	182	24 (13.2)	158 (86.8)	Ref		Ref	
General hospital	161	21 (13)	140 (87.0)	1.01 (0.54–1.90)	0.69	1.16 (0.59–2.27)	0.67
Tertiary care/Specialty hospital	95	9 (9.5)	86 (90.5)	1.45 (0.65–2.36)	0.37	1.98 (0.84–3.67)	0.17
HCWs category							
Practitioner (Medical and Dental)							
Nursing and Midwifery	176	23 (13.1)	153 (86.9)	Ref		Ref	
Lab technicians	127	12 (9.4)	115 (90.6)	1.44 (0.69–2.02)	0.33	1.01 (0.88–1.34)	0.68
Others	68	9 (13.2)	59 (86.8)	0.86 (0.68–1.61)	0.71	0.61 (0.46–1.24)	0.26
	67	10 (14.9)	57 (85.1)	0.99 (0.43–2.23)	0.97	0.85 (0.35–2.06)	0.72
Work experience							
Less the 5 years	131	16 (12.2)	115 (87.8)	Ref		Ref	
5 to 10 years	157	21 (13.4)	136 (86.6)	0.90 (0.45–1.81)	0.77	1.36 (0.58–2.22)	0.48
>10 years	150	17 (11.3)	133 (88.7)	1.09 (0.53–2.25)	0.82	2.86 (0.81–4.04)	0.1
Smoking status							
Non-smoker	241	34 (14.1)	207 (85.9)	Ref		Ref	0.028 ^**^
Daily/Rarely	197	20 (10.2)	177 (89.8)	1.45 (0.81–2.62)	0.21	2.16 (1.09–3.29)	
Sleeping hours per day (mean ± SD)	6.9 ± 1.4			1.36 (1.08–1.70)	0.008 ^**^	1.35 (1.07–1.70)	0.013 ^**^

^*^ Variable(s) entered for logistic regression analysis: age, gender, marital status, nationality, work setting, HCWs category, work experience, smoking status, sleep hours per day, ^**^ *p* value less than 0.05 was considered as statistically significant.

## Data Availability

The data presented in this study are available on request from the corresponding author.
